# Anti-NMDAR Encephalitis: Higher Suspicious Needed for Earlier Diagnosis (Case Report, Literature Review and Diagnostic Criteria)

**DOI:** 10.1155/2019/7476254

**Published:** 2019-12-28

**Authors:** Chanaka Amugoda, Noushin Chini Foroush, Hamed Akhlaghi

**Affiliations:** ^1^Emergency Department, Werribee Mercy Hospital, Melbourne, Australia; ^2^Medical Registrar, Austin Health, Melbourne, Australia; ^3^Emergency Physician, St. Vincent's Hospital, University of Melbourne, Melbourne, Australia

## Abstract

**Background:**

Auto-immune mediated anti-NMDA receptor encephalitis is a very common delayed diagnosed encephalitis which predominately affecting young population.

**Objectives:**

This encephalitis is relatively unknown amongst emergency physicians and a majority of patients are admitted to psychiatric wards before their diagnosis is confirmed and appropriate treatments are commenced. We reported a case of a 22-year-old female presented to our emergency department with acute psychiatric symptoms. She was initially diagnosed with first presentation of acute psychosis and was hospitalised under mental health act. further assessment in the emergency department identified possible an organic cause for her acute psychosis and she was later admitted under medical team after her mental health assessment order was revoke. Several days later, her CSF result was positive with anti-NMDA receptor anti-bodies. Appropriate treatments were instituted leading to her full recovery.

**Conclusion:**

This case was the first confirmed anti-NMDA receptor encephalitis in our emergency department. It highlights the importance of thorough assessment of psychiatric presentations to emergency departments and consideration of auto-immune medicated encephalitis as one of the differential diagnosis in young patients presenting with first acute psychotic episode.

## 1. Introduction

Encephalitis causes significant morbidity and mortality worldwide. Encephalitis is defined by the Consensus Statement of the *International Encephalitis Consortium* as severe Inflammation of the brain parenchyma associated with debilitating neurologic dysfunction [1]. Traditionally, viral encephalitis was the most recognisable type of encephalitis. However, in the last 15 years with advancements in medical imaging and the development of new neurologic biomarkers, other noninfectious, mainly autoimmune-mediated encephalitis have been identified and reported [2]. Anti-N-methyl-D-aspartate receptor (NMDAR) encephalitis was first described by Dalmau et al. in 2005 [3]. NMDAR encephalitis is a common cause of autoimmune encephalitis [4] frequently misdiagnosed by the treating physician as a psychiatric illness [5]. In this article, we review the clinical presentation, investigation and diagnosis of NMDAR encephalitis in conjunction with a case report. The article concludes with proposed diagnostic criteria for NMDAR encephalitis, developed to assist primary care and emergency physicians should they suspect a possible diagnosis of NMDAR encephalitis.

## 2. Clinical Presentation

The clinical presentation typically progresses in four stages: the prodromal phase, the psychotic phase, the unresponsive phase and the hyperkinetic phase [6]. During the prodromal phase, patients experience unspecific viral-like symptoms such as low-grade fever, headache, upper respiratory tract symptoms, fatigue, nausea, vomiting and diarrhoea. However, fever and headache more commonly occur after the onset of neuropsychiatric symptoms. The initial phase manifests in 70 to 86 per cent of patients and can last up to 21 days [6, 7]. The psychotic features typically manifest within two weeks following the prodromal phase. Majority of patients seek medical attention during this phase with symptoms of agitation, paranoid delusions, auditory and visual hallucinations, bizarre behaviour, mood liability, insomnia, depression, anxiety, disorganised thoughts, epileptic seizures, cognitive impairment, and memory deficit [7, 8]. Frequently half of NMDAR encephalitis patients is misdiagnosed predominantly due to psychotic features [6]. A seizure is commonly observed in up to 82 per cent of patients. The main manifestations of the unresponsive phase are mutism and akinesia followed by hyperkinetic phase. Patients experience autonomic instability, hypo- or hypertension, hypo- or hyperthermia, cardiac arrhythmia and hypoventilation [6]. In cases with severe hypoventilation, ventilatory support may be required.

Younger patients mainly present with behavioural disturbances instead of frank psychosis hindering the diagnosis of anti-NMDAR encephalitis in children. Frequently they present with nonpsychiatric manifestations such as seizures, dystonia or mutism [9]. In contrast, psychiatric symptoms and memory deficit are the main manifestation of the disease amongst patients over 44 years old [10].

### 2.1. Disease Pathogenesis

Anti-N-methyl-D-aspartate receptor, mainly found in the forebrain, hippocampus and limbic system, is a tetrameric complex composed of two GluN1 subunits and combination of two GluN2 or GluN3 subunits. The function of this receptor has been linked to learning, memory, cognition, and behaviour [6]. Current evidence suggests that IgG antibodies in the serum and CSF bind specifically to the GluN1 subunit are the cause of disease pathogenesis [8].

Higher incidence of teratoma and post-HSE (herpes-simplex-encephalitis) NMDAR encephalitis suggests malignancies and infections as triggers for this disease. A recent study shows ovarian teratoma contains abnormal CNS neuron leading to extra-axial expression of NMDA receptor, however in 80% of anti-NMDAR encephalitis cases, no tumour is found [11]. Due to correlation between HSE and NMDAR encephalitis, it is suggested that to test Anti-NMDAR antibodies in CSF of patients with relapse post-HSE [2]. Almost 90% of patients with anti-NMDAR encephalitis manifest the prodromal phase, support the idea of infective aetiologies. Nevertheless, extensive CSF sampling and brain biopsies fail to identify direct viral pathogenesis. The underlying pathophysiology of the prodrome phase is unclear, it is uncertain whether it is solely an early manifestation of immune-mediated response or an infection interrupting the normal blood-brain barrier function letting antibodies to cross [12].

### 2.2. Epidemiology

Anti-NMDAR encephalitis is a female predominant disease, as women represent up to 80 per cent of reported cases [9]. It is also considered a middle-aged person disease, although anti-NMDAR encephalitis has been reported in cases of people aged between two months and 90 years [9, 10, 13]. The male : female ratio is as high as 40 per cent amongst patients over 44 years old [13].

Teratoma is the most common tumour associated with this disease and around 40–50 per cent of female patients with anti-NMDAR encephalitis have been reported to have teratoma. A large series study revealed that 98 per cent of tumours are ovarian teratomas [9]. Detection of other malignancies such as neuroblastoma or Hodgkin's lymphoma is rare [9]. Underlying malignancies are less common in younger or male patients.

Paediatric patients (younger than 18 years old) have a higher incidence of seizures as the first presentation for medical attention compared to adult patients. They have more favourable outcome and less likely to have CSF pleocytosis [14]. The presence of underlying tumour is less common in the paediatric population and in females more likely to be an ovarian teratoma if there is a tumour (90%) [14].

### 2.3. Diagnosis

Diagnosis of anti-NMDAR encephalitis is based on the presentation of NMDAR antibodies in CSF or serum. The NMDAR antibodies in CSF have higher specificity and sensitivity in comparison to the serum antibodies. Antibodies in CSF are always present at the time of diagnosis nevertheless, to avoid false-negative or false-positive results, it is recommended to test NMDAR antibodies in both CSF and serum [15].

The titre of NMDAR antibodies increases with disease progression and there is a direct correlation between the severity of the disease, clinical presentation, and underlying malignancies with antibody titration [12]. Other abnormal nonspecific findings in CSF are pleocytosis (60–76%), high protein (19%), oligoclonal band (17%) and increased opening pressure in nearly 40% [3, 16, 17]. Glucose and chloride concentrating of CSF is almost always normal [17]. A recent study proposed CSF cell-free mitochondrial DNA as a potential biomarker in anti-NMDAR encephalitis [18], however it takes several years to get into general practice.

Imaging of the brain, although not a widely accepted approach, is mainly requested as part of the initial clinical evaluation of the first episode of acute psychiatric illness or to exclude other causes of acute confusion [19]. Only one-third of patients with anti-NMDAR encephalitis have positive brain imaging results, with changes reported in hippocampi, cerebellar and cerebral cortex, fronto-basal and insular regions, basal ganglia, brainstem or spinal cord [3, 6, 12]. These imaging findings are nonspecific and have poor correlation with disease severity and symptoms and fully resolved after treatment [6]. Recent MRI study showed only half of anti-NMDAR encephalitis showed abnormalities in their brain regions including medial temporal lobe, frontal lobe subcortical white matter and periventricular region [20]. Therefore, the imaging modalities are not a useful and recommended clinical tool in diagnosis of anti-NMDAR encephalitis.

PET scans (FDG-PET) have been suggested as a potential biomarker in diagnosis of autoimmune encephalitis and showed hypometabolism of the occipital lobe in anti-NMDAR encephalitis [6, 21].

EEG findings are abnormal in 90% of patients with anti-NMDAR encephalitis [12, 16]. The most frequent abnormal EEG finding in a series of 100 patients was delta-theta wave which is described as slow-wave with or without epileptic features. In up to the third of anti-NMDAR encephalitis patients, the EEG pattern shows distinct EDB (extreme delta brush) pattern independent of body Circadian rhythm which can lead to diagnosis of the disease in the early stages [22]. Due to higher incidence of malignancies in anti-NMDAR encephalitis, screening with CT, MRI, abdominal and transvaginal ultrasound is recommended.

### 2.4. Treatment and Outcome

The mainstay of current treatment is conservative management, high-dose corticosteroid, intravenous immunoglobulins (IVIg), plasma exchange (PE), immunotherapy, and tumour removal if present. The first line therapy compromises of corticosteroid, IVIg, and PE with or without tumour removal. Recovery in four weeks has been observed in approximately 50 per cent of patients. The second line therapy consists of immunotherapy with rituximab or cyclophosphamide and is used if patient relapses or the first-line therapy fails.

Currently, there is no high-quality controlled trial evidence on the standard and optimal therapeutic guideline for anti-NMDAR encephalitis. The class IV expert opinion is to start a combination of IVIg and methylprednisolone for five days as the first line, and if there is no clinical improvement after 15 days then the second-line therapy should commence which consists of rituximab with monthly cycle of cyclophosphamide [23].

Patients with no underlying malignancy have shown a higher rate of relapse and resistance to usual treatment, less favourable outcome and prolonged recovery time [16]. The early immunosuppressive therapy and early resection of the tumour are associated with improved outcome and recovery [12, 24, 25]. The intra-thecal treatment has been reported in a case series resistant to first-line and second-line therapies [26]. Symptomatic treatment with lorazepam and ECT for management of catatonia has been reported with efficacy between 80 and 90 per cent [6, 27].

Anti-NMDAR encephalitis tends to have a better outcome in comparison with other autoimmune medicated encephalitis with a full or substantial recovery rate of 80–90 per cent after 24 months [14, 16]. Mortality is around six per cent, and higher mortality rates have been observed amongst older patients or patients with significant CSF pleocytosis at the time of diagnosis [14]. In a case series of 501 patients, early treatment, low severity of symptoms in the early phase and no need for ICU admission, were associated with better outcomes and fewer relapses [16]. Improvement of symptoms is observed after a few weeks post commencement of treatment. However full recovery may take up to three years to return to baseline functioning [6]. Structural hippocampal damage and cognitive deficits such as impairment in attention, working memory, episodic memory and executive function comprise the major burden and long-term morbidity of anti-NMDAR encephalitis [28].

## 3. Case Report

A 28-years-old female was brought into the hospital by her husband concerned by his wife's recent bizarre and abnormal behaviour in the setting of recent university exam and work-related stress. Her main symptoms were acute behavioural changes for five days including lack of sleep, paranoia, talkative, labile mood, and auditory hallucination. The woman is a PhD student who is bilingual in English and Chinese. Her husband reported that she was hearing voices and has become paranoid. Although his wife was fluent in English, she had begun to communicate in her native language. The patient complained of a headache for one week for which she was taking therapeutic dose of paracetamol. She did not have any recent febrile illness, trauma, or head injury. The patient has no history of psychiatric illness or illicit drugs use. She was not on any regular medication, a nonsmoker, and social alcohol drinker.

In the emergency department, the patient presented as paranoid and confused. Vital signs showed intermittent tachycardic with HR between 80 and 150/min and hypertensive with BP 140/80 mmHg. The patient was initially afebrile, and her initial investigations showed only slight elevation of white cell count up to 15 ∗ 10^6^ mmol/L with significant neutrophilia. The rest of the clinical investigations including electrolytes, renal function, liver function, C-reactive protein, urine test, thyroid function test and CT Brain was normal. A neurological examination was completely normal with no signs of meningitis. Patients GCS was fluctuating from 14 to 15. During her emergency stay, she became agitated requiring both oral diazepam 5 mg and intravenous Droperidole 5 mg for sedation. The patient was referred to the psychiatric team and accepted for further management and treatment for the first episode of psychosis.

While the patient was in the emergency department, she became more confused and agitated, and her temperature spiked to 38.5°C. A lumbar puncture was conducted. Her CSF results indicated elevated protein 0.59 gr/L and white cell count of 200 with 13 of polymorphs and 187 mononuclear cells. There were no bacteria seen in the CSF sample (Table 1). The patient was diagnosed with possible infective encephalitis and commenced treatment with intravenous ceftriaxone 1gr/daily and acyclovir to cover for possible HSE was commenced. The previous admission to the psychiatric unit was changed to medical admission.

During her admission under the medical team, she underwent Brain MRI study which was reported normal. Her varicella-zoster and herpes PCR of CSF came back negative alongside with autoimmune panel tests. Her GCS continued to drop to as low as five, and she became verbally unresponsive and more catatonic despite being on intravenous antibiotics.

After one week in conjunction with a neurology input, the diagnosis of autoimmune encephalitis and possible anti-NMDAR encephalitis was suspected, and pelvic ultrasound was requested which showed a 25 ∗ 100 ∗ 90 mm large multiocular cyst with no vascularity with no solid material suggestive of teratoma. At this point, the patient commenced on intravenous immunoglobulin, methylprednisone and rituximab. During this time the patient underwent laparotomy and a subsequent right-side oophorectomy and was admitted to ICU due to low GCS. Her length of stay in ICU was twenty days. Her admission was complicated with one episode of self-remitting generalised tonic-clonic seizure, autonomic dysfunction and orofacial dyskinesia requiring Botox treatment. The patient's EEG result was consistent with a moderate diffuse encephalopathy, and 24H EEG showed diffuse delta brush wave highly suggestive of anti-NMDAR encephalitis. A subsequent CSF test was positive with NMDAR antibody confirming diagnosis as anti-NMDAR encephalitis.

Upon discharge, the patient needed ongoing cognitive rehabilitation. She was subsequently transferred to rehabilitation centre where she was able to progress positively in terms of behaviour and cognition, returning to pre-morbid functional level and activities and since has not experienced any relapse in symptoms.

## 4. Discussion

This case report presented a delayed diagnosis of anti-NMDAR encephalitis with most of the typical features and ovarian teratoma. Lack of clinical suspicion and the absence of diagnostic criteria were two important factors that circumvent earlier diagnosis.

This case showed the complexity of patient symptoms and nonspecific nature of anti-NMDAR encephalitis. Our case was referred to the psychiatric unit for treatment of first psychosis because the initial investigations failed to reveal an organic cause for the patient's presentations. Even after abnormal CSF, the patient was diagnosed with possible viral or bacterial encephalitis. The alternative diagnosis of auto-immune encephalitis and anti-NMDAR encephalitis was considered when the patient failed to respond to initial treatment and her level of consciousness continued to decline.

Diagnostic criteria for autoimmune encephalitis depend on detection of autoantibodies and response to immunotherapy which are not available at the time of presentation to emergency department or early clinical evaluation. Besides, the capability for detection of the antibody may not be readily available, and in most cases, it takes several weeks for the result to become available which potentially delays the diagnosis while early treatment of anti-NMDAR encephalitis is associated with more favourable outcome. The following clinical diagnostic approach was proposed by a group of neurologists to assist with early diagnosis and treatment of anti-NMDAR encephalitis [2].

### 4.1. Diagnostic Criteria for Probable Anti-NMDAR Encephalitis [2]

- Rapid onset (less than three months) of at least four of the six following major groups of symptoms (or three at the presence of underlying tumour such as teratoma):
  1. Abnormal (psychiatric) behaviour or cognitive dysfunction.
  (2) Speech dysfunction (pressured speech, verbal reduction, mutism).
  (3) Seizure.
  (4) Movement disorder, dyskinesias or rigidity/abnormal postures.
  (5) Decreased level of consciousness.
  (6) Autonomic dysfunction or central hypoventilation.
- At least one of the following laboratory study results:
  1. Abnormal EEG (focal or diffuse slow or disorganised activity, epileptic activity, or extreme delta brush).
  2. CSF with pleocytosis or oligoclonal bands.
- Reasonable exclusion of other disorders such as CNS infection, herpes simplex virus encephalitis, epileptic disorders, or known psychiatric disease.

Memory testing is not part of the criteria due to poor accuracy for memory assessment during acute psychosis, abnormal behaviours or agitation. It is recommended to start immunotherapy once the criteria are met whilst checking for underlying malignancies or tumour [2].

The proposed diagnostic criteria have high sensitivity of 90 per cent and specificity of 96 per cent. However, duration between onset of symptoms until symptoms could fulfil the proposed diagnostic criteria could take up to two weeks [29].

The patient exhibited acute behavioural changes accompanied by autonomic dysfunction, decreased level of consciousness, speech difficulty and cognitive disturbances. Using the criteria, our patient could have been diagnosed as possible anti-NMDAR encephalitis, and definitive treatment could have commenced sooner.

It is important to increase awareness of primary care physicians and emergency doctors of possible organic and auto-immune aetiology for psychiatric presentations and share the understanding that normal initial investigations do not completely exclude organic causes. A high level of suspicious and detailed history taking is required to consider an alternative diagnosis such as anti-NMDAR encephalitis.

Therefore, once a patient (particularly those aged in their twenties and thirties), presents to the emergency department or primary care physician with acute onset psychiatric symptoms or seizure while manifesting four of the six criteria, CSF testing for pleocytosis or oligoclonal bands should be urgently performed. In most hospitals, the results can be available within a couple of hours. If in-house an EEG service is available, the abnormal EEG findings can confirm the diagnosis and prevent significant delays in commencement of the immunotherapy.

## Figures and Tables

**Table 1:** CSF result of the patient.

	Tube 1	Tube 2	Tube 3
Erythrocyte (∗ 10^6^/L)	660	1940	7020
White cell (∗ 10^6^/L)	200	380	290
Polymorph (∗ 10^6^/L)	13	30	35
Mononuclear (∗ 10^6^/L)	187	350	255
